# Editorial: New frontiers in HIV antiretroviral treatment: from the management of metabolic complications and chronic inflammation to new long-acting regimens

**DOI:** 10.3389/fmed.2024.1402565

**Published:** 2024-04-03

**Authors:** Salvatore Martini, Guido Poli

**Affiliations:** ^1^Division of Infectious Diseases, University of Campania “Luigi Vanvitelli”, Naples, Italy; ^2^Division of Immunology, Transplantation and Infectious Diseases, Università Vita-Salute San Raffaele, IRCCS San Raffaele Scientific Institute, Milan, Italy

**Keywords:** HIV, inflammation, intima media thickness (IMT), antiretroviral (ARV) therapy, long-acting (LA) HIV therapy

Aim of this Research Topic is the evaluation of new parameters linked to anti-retroviral therapy (ART), no longer focused exclusively on its viro-immunological efficacy—the main target of every approved regimen—but also on the analysis of the long-term metabolic impact and pressure on residual viremia. It is important, in fact, to achieve a stable containment of HIV replication avoiding residual low-level replication, that, although not crucial in immediate clinical terms, it has been linked to release of pro-inflammatory cytokines associated to endothelial dysfunction and development of cardiovascular disease (CVD) that worsens long-term prognosis of people living with HIV (PLWH) (1).

The development of atherosclerosis is a complex process involving endothelial dysfunction and arterial inflammation. Systemic inflammation may exacerbate atherogenesis as it has been documented with HIV infection. This condition in fact causes a state of persistent inflammation that may induce early biologic aging and higher risk of CVD (1). In response to proatherogenic stimuli, the endothelium alters production of nitric oxide, which affects vascular tone, increases permeability, allowing entry of lipids, and releases chemokines. These effects cause a thickening of the tunica intima that may progress into a fibroatheroma, characterized by intraplaque calcifications and the overlying fibrous cap. Some fibrous caps are vulnerable to erosion or rupture, resulting in thrombus formation and potentially myocardial infarction (MI), stroke, or sudden cardiac death. Viral suppression with ART alleviates but does not normalize the inflammatory profile associated with HIV infection. In a recent study, we provided evidence of increased pathological intima media thickness and atheromatous plaques in patients with HIV infection, treated with protease inhibitors (2). In this regard, many epidemiologic studies in PLWH have demonstrated an increased risk of MI and stroke even after exclusion of traditional CVD risk factors (3).

These unconventional parameters are of further relevance in the context of new therapy schemes in which ART is not administered orally but by intramuscular injection leading to a half-life of ca. 60 days. This long-acting regimens represents a recent, relevant innovation in the field of anti-HIV therapy that may guarantee a better quality of life at least for some patients. It is, therefore, important to evaluate whether the pharmacokinetics determined by stable drug release will reduces the possible incidence of residual viremia and long-term inflammation. At the same time, the metabolic tolerability and effects on weight gain of Cabotegravir will need to be evaluated.

As part of the topic, a review by Lazarus et al. evaluated the various trials of long-acting regimens in the context of pre-exposure prophylaxis (PREP). This is a further new frontier of application of long-acting regimes that can be particularly effective, allowing a long period of coverage and prevention from infection for those on PREP. Long-acting regimens should improve PREP adherence as efficacy would not be related to continuous or “on demand” oral schemes. Another variable that should be considered is related to the observation that individuals who adhere to PREP may also use narcotic substances and/or alcohol during unprotected sexual acts, a habit that may affect the efficacy of oral PREP. The adoption of long-acting PREP regimens should solve this problem by maintaining a stable concentration of drugs in blood without requiring oral intake on days of risky sexual intercourses. The review article discusses the safety and pharmacokinetic validity of this regimen in the trials examined.

An original study by Ma et al. investigated the results of patients undergoing monitored ART suspension with restarting drug administration within 16 weeks from interruption. Voluntary suspensions of drugs with poor adherence to therapy are quite frequent in PLWH on treatment, particularly in some geographical areas with poor socio-economic conditions and in patients of young age. The study shows that although retention in care within 16 weeks is not related to significant harm, nonetheless, there is need of optimizing adherence to avoid worsening of clinical conditions with onset of comorbidities and emergence of ART resistance mutations. The authors also underscore that no more than 16 weeks should pass from the voluntary suspension of ART and that, at the same time, clinicians should underline the importance of adherence for long-term success to their patients undergoing ART suspension. Once again, the adoption of long-acting ART regimens should be a crucial asset to optimize adherence, provided that patients respect the timing of the injections, to avoid the loss of effectiveness of the treatment and the appearance of cross-resistance.

Another study by Raquel Martín-Iguacel et al. evaluated the risk of heart disease in HIV late presenters compared to those who did not share this condition. The poorer clinical conditions of these patients are often related to higher inflammatory parameters that could cause a higher CV risk. However, the study shows that if there are no previous CV pathologies, late presenters do not show a greater risk of events than the control arm. As it is appropriate, in any case, to improve the prevention of CV risk in PLWH, long-acting regimens should guarantee stable control of virus replication and reduce inflammatory parameters often associated with the worse long-term prognosis in late presenters vs. their controls.

Another study by Zhang et al. evaluated the efficacy and tolerability of ainuovirine (ANV), a novel non-nucleoside reverse transcriptase inhibitor, compared to efavirenz (EFV)-based regimen. ANV was non-inferior to EFV in terms of efficacy, showing an additional positive impact on reducing dyslipidemia. This research is in line with the search for new ART regimens that are not only effective in blocking HIV replication, but also show a favorable long-term metabolic profile.

In conclusion, ART is constantly evolving with the goal of achieving long-term control of HIV replication with effective, well-tolerated regimens that respect patients' quality of life. The adoption of two drug regimens with long-acting formulations represent a new frontier of ART fulfilling these goals. Future studies will allow a better understanding of whether the different pharmacokinetics associated with long-acting regimens will result in terms of stable suppression of viral replication, better compliance and reduced inflammatory profile thereby ensuring reduced comorbidities and fewer long-term clinical complications.

## Author contributions

SM: Conceptualization, Writing – original draft, Writing – review & editing. GP: Supervision, Writing – review & editing.
